# Delineation of Steroid-Degrading Microorganisms through Comparative Genomic Analysis

**DOI:** 10.1128/mBio.00166-16

**Published:** 2016-03-08

**Authors:** Lee H. Bergstrand, Erick Cardenas, Johannes Holert, Jonathan D. Van Hamme, William W. Mohn

**Affiliations:** a Department of Microbiology and Immunology, Life Sciences Institute, University of British Columbia, Vancouver, British Columbia, Canada; b Department of Biological Sciences, Thompson Rivers University, Kamloops, British Columbia, Canada

## Abstract

Steroids are ubiquitous in natural environments and are a significant growth substrate for microorganisms. Microbial steroid metabolism is also important for some pathogens and for biotechnical applications. This study delineated the distribution of aerobic steroid catabolism pathways among over 8,000 microorganisms whose genomes are available in the NCBI RefSeq database. Combined analysis of bacterial, archaeal, and fungal genomes with both hidden Markov models and reciprocal BLAST identified 265 putative steroid degraders within only *Actinobacteria* and *Proteobacteria*, which mainly originated from soil, eukaryotic host, and aquatic environments. These bacteria include members of 17 genera not previously known to contain steroid degraders. A pathway for cholesterol degradation was conserved in many actinobacterial genera, particularly in members of the *Corynebacterineae*, and a pathway for cholate degradation was conserved in members of the genus *Rhodococcus*. A pathway for testosterone and, sometimes, cholate degradation had a patchy distribution among *Proteobacteria*. The steroid degradation genes tended to occur within large gene clusters. Growth experiments confirmed bioinformatic predictions of steroid metabolism capacity in nine bacterial strains. The results indicate there was a single ancestral 9,10-seco-steroid degradation pathway. Gene duplication, likely in a progenitor of *Rhodococcus*, later gave rise to a cholate degradation pathway. *Proteobacteria* and additional *Actinobacteria* subsequently obtained a cholate degradation pathway via horizontal gene transfer, in some cases facilitated by plasmids. Catabolism of steroids appears to be an important component of the ecological niches of broad groups of *Actinobacteria* and individual species of *Proteobacteria*.

## IMPORTANCE

Steroids are ubiquitous growth substrates for environmental and pathogenic bacteria, and bacterial steroid metabolism has important pharmaceutical and health applications. To date, the genetics and biochemistry of microbial steroid degradation have mainly been studied in a few model bacteria, and the diversity of this metabolism remains largely unexplored. Here, we provide a bioinformatically derived perspective of the taxonomic distribution of aerobic microbial steroid catabolism pathways. We identified several novel steroid-degrading bacterial groups, including ones from marine environments. In several cases, we confirmed bioinformatic predictions of metabolism in cultures. We found that cholesterol and cholate catabolism pathways are highly conserved among certain actinobacterial taxa. We found evidence for horizontal transfer of a pathway to several proteobacterial genera, conferring testosterone and, sometimes, cholate catabolism. The results of this study greatly expand our ecological and evolutionary understanding of microbial steroid metabolism and provide a basis for better exploiting this metabolism for biotechnology.

## INTRODUCTION

Microbial steroid degradation is an important process in several ways. Steroids constitute a highly abundant class of organic molecules in natural environments. Sterols are a major constituent of the membranes of all eukaryotic cells (1). Other steroids such as bile salts and steroid hormones are excreted into the environment by vertebrates. Thus, during decomposition of biomass and excreta, these steroids are available to microorganisms as significant potential growth substrates. Bacterial steroid degradation is also medically relevant, as a functioning cholesterol degradation pathway is essential for the survival of phagocytized *Mycobacterium tuberculosis*, the pathogen behind the tuberculosis epidemic (2). And, in the pharmaceutical industry, bacterial steroid biotransformation has been explored as a source of biocatalysts for the production of steroid-based drugs (3).

**FIG 1:**
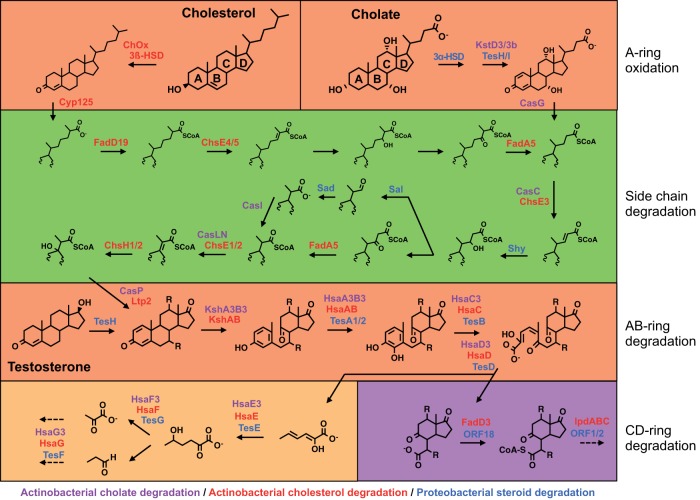
Aerobic 9,10-seco degradation pathways for cholesterol, cholate, and testosterone. The steroid ring structure is degraded by oxygen-dependent opening and subsequent hydrolytic cleavage of rings A and B. Subsequent degradation of the C and D rings occurs by a mechanism not yet described. In *Actinobacteria*, side chain degradation and ring opening can occur simultaneously. Characterized or annotated enzymes involved in the degradation of cholesterol by *Actinobacteria* are red, those involved in the degradation of cholate by *Actinobacteria* are lilac, and those involved in the degradation of testosterone or cholate by *Proteobacteria* are blue. Protein nomenclature is based on that of *Rhodococcus jostii* RHA1, *Mycobacterium tuberculosis* H37Rv, *Comamonas testosteroni* TA441, and *Pseudomonas* sp. strain Chol1, and not all proteins are named.

Knowledge of steroid catabolism pathways is based mainly on the investigation of a few *Actinobacteria* and *Proteobacteria* species, which have been shown to grow on and, in several cases, mineralize steroids (4–10). Testosterone degradation and bile salt degradation have been best studied in *Comamonas testosteroni* strains CNB-2 and TA441 (4) and *Pseudomonas* sp. strain Chol1 (11). Cholesterol degradation has been best studied in *Rhodococcus jostii* RHA1 (9) and *Mycobacterium tuberculosis* H37Rv (12–14). Recently, bile salt degradation has also been studied in strain RHA1 (8). In all of these cases, degradation of the steroid nucleus follows very similar progressions, using the 9,10-seco pathway (Fig. 1). Where present, side chains are degraded by a beta-oxidation process. In the case of cholesterol, the alkyl side chain is initially activated by a monooxygenase (15–17). Bacterial steroid uptake is poorly understood. *Actinobacteria* spp. appear to take up cholesterol with a complex ABC transporter comprised of many proteins (18). The genes encoding steroid catabolism that have been identified tend to occur in large clusters encoding major components of the degradation pathways (see Fig. S1 in the supplemental material). In the cholesterol pathway, the genes encoding C and D ring degradation are in a distinct regulon (19–21). It is not known how well these characterized pathways represent steroid catabolism in other microorganisms.

Several denitrifying *Proteobacteria* spp. have been described to degrade cholesterol and testosterone under anaerobic conditions (22–24) using dioxygen-independent reactions to degrade the steroidal core (25, 26), in contrast to the aerobic 9,10-seco pathway. Unfortunately, the genetic and biochemical background of the anaerobic steroid degradation pathway is largely unknown, and genome sequences are not available for these bacteria.

Given the ubiquity of steroids in the natural environment, it is conceivable that diverse microbial taxa possess steroid degradation capabilities and occupy a range of ecological niches. One expedient, culture-independent method of discovering new steroid-degrading taxa is mining genomic databases for steroid degradation gene clusters homologous to those found in known steroid degraders. Although biased toward medically or economically important organisms, genome databases now represent diverse microbial taxa. Thus, analysis of genomes can potentially determine the occurrence of biochemical pathways among taxa and may lend insight into the evolution of these pathways. In this study, we explored the distribution of pathways homologous to known steroid degradation pathways among genomes in the curated RefSeq database hosted by the National Center for Biotechnology Information. All fungal, archaeal, and bacterial genomes from RefSeq were searched using hidden Markov models (HMMs), and a subset was searched using reciprocal BLAST. The results were used to infer the distribution of the pathways among taxa and to deduce aspects of the evolution of the pathways. Several newly identified steroid degraders were tested *in vitro* to validate predictions of their steroid degradation capacities.

## RESULTS

### Identification of steroid-degrading organisms.

Hidden Markov models (HMMs) were used to search sequenced microbial genomes in order to identify putative steroid-degrading organisms. A total of twenty-five HMMs were used, representing variants of eight key enzymes involved in steroid nucleus degradation (Table 1), to search 8,277 complete and draft bacterial, archaeal, and fungal genomes downloaded from RefSeq. A total of 265 putative steroid-degrading organisms were identified, their genomes encoding at least six of the eight key enzymes, including KshA/CtCNB1_1306, the oxygenase subunit of the 3-ketosteroid-9α-hydroxylase, and HsaC/TesB, an extradiol dioxygenase required for A/B ring degradation. The organisms identified were *Actinobacteria* spp. as well as alpha-, beta-, and gammaproteobacteria (see Table S1 in the supplemental material). The putative steroid-degrading bacteria were mainly from soil, eukaryotic host, and marine environments, with the majority of host-associated ones being pathogens (see Fig. S2). No putative steroid-degrading fungi or *Archaea* were detected.

**TABLE 1:** Table listing eight orthologous groups of proteins from *Rhodococcus jostii* RHA1, *Mycobacterium tuberculosis* H37Rv, and *Comamonas testosteroni* CNB-2 used as references for HMM generation^a^

RHA1/H37Rv name	CNB-2 name	CNB-2 testosterone/cholate cluster	RHA1cholate cluster	RHA1cholesterol cluster	H37Rvcholesterol cluster	Annotation
KstD	TesH	CtCNB1_1357	RHA1_ro05798	RHA1_ro04532	Rv3537	3-Ketosteroid-dehydrogenase
		(CTCNB1_RS06925)	(RHA1_RS28305)	(RHA1_RS22090)		
			RHA1_ro05813			
			(RHA1_RS28380)			
KshA	No name	CtCNB1_1306	RHA1_ro05811	RHA1_ro04538	Rv3526	3-Ketosteroid-9-alpha hydroxylase (oxygenase)
		(CTCNB1_RS06665)	(RHA1_RS28370)	(RHA1_RS22120)		
HsaA	TesA2	CtCNB1_1356	RHA1_ro05802	RHA1_ro04539	Rv3570c	3-Hydroxy-9,10-seconandrost-1,3,5(10)-triene-9,17-dione-4-hydroxylase (oxygenase)
		(CTCNB1_RS06920)	(RHA1_RS28325)	(RHA1_RS22125)		
HsaC	TesB	CtCNB1_1275	RHA1_ro05803	RHA1_ro04541	Rv3568c	3,4-Dihydroxy-9,10-secoandrosta-1,3,5(10)-triene-9,17-dione-4,5-dioxygenase
		(CTCNB1_RS06510)	(RHA1_RS28330)	(RHA1_RS22135)		
HsaD	TesD	CtCNB1_1354	RHA1_ro05797	RHA1_ro04540	Rv3569c	4,5-9,10-Diseco-3-hydroxy-5,9,17-trioxoandrosta-1(10),2-diene-4-oate hydrolase
		(CTCNB1_RS06910)	(RHA1_RS28300)	(RHA1_RS22130)		
HsaE	TesE	CtCNB1_1353	RHA1_ro05799	RHA1_ro04533	Rv3536c	2-Hydroxyhexa-2,4-dienoate hydratase
		(CTCNB1_RS06905)	(RHA1_RS28310)	(RHA1_RS22095)		
HsaF	TesG	CtCNB1_1351	RHA1_ro05801	RHA1_ro04535	Rv3534c	4-Hydroxy-2-oxohexanoate aldolase
		(CTCNB1_RS06905)	(RHA1_RS28320)	(RHA1_RS22105)		
HsaG	TesF	CtCNB1_1352	RHA1_ro05800	RHA1_ro04534	Rv3535c	Propanol dehydrogenase
		(CTCNB1_RS06900)	(RHA1_RS28315)	(RHA1_RS22100)		

a Locus tags of the respective proteins are listed as used in this study and after subsequent GenBank reannotation (the latter are indicated in parentheses).

A total of 212 putative steroid-degrading *Actinobacteria* spp. were identified, representing 16 genera. These included most genera in the suborder *Corynebacterineae* (*Amycolicicoccus*, *Dietzia*, *Gordonia*, *Mycobacterium*, *Nocardia*, *Rhodococcus*, and *Tsukamurella*) as well as the genera *Actinoplanes*, *Aeromicrobium*, *Amycolatopsis*, *Arthrobacter*, *Nocardioides*, *Saccharomonospora*, *Salinispora*, *Streptomyces*, and *Thermomonospora* (see Table S1 in the supplemental material). With few exceptions, all available genomes from these genera appear to encode at least one steroid catabolism pathway. The exceptions lacking such pathways were *Rhodococcus* sp. strain AW25M09 (affiliated with *R. fascians*), all draft and complete genomes of *Mycobacterium leprae*, 5 of 6 draft and complete genomes of *Saccharomonospora* spp., 3 of 4 complete genomes of *Actinoplanes* spp., and 55 of 57 draft and complete genomes of *Streptomyces* spp*.*

A total of 53 putative steroid-degrading *Proteobacteria* spp. were identified. These were individual species within the genera *Burkholderia*, *Comamonas*, *Cupriavidus*, *Glaciecola*, *Hydrocarboniphaga*, *Marinobacterium*, *Novosphingobium*, *Pseudoalteromonas*, *Pseudomonas*, *Shewanella*, and *Sphingomonas* (see Table S1 in the supplemental material). They also included unclassified species of the alphaproteobacteria and gammaproteobacteria as well members of the SAR86 clade of gammaproteobacteria and the OM60 clade of the oligotrophic marine *Gammaproteobacteria* (OMG) group. Notably, genomes of *Glaciecola*, *Marinobacterium*, *Pseudoalteromonas*, and *Shewanella* and of OM60 and SAR86 all represent organisms from marine environments. In contrast to the actinobacterial genera, only one or a few genomes from each proteobacterial genus appear to encode steroid catabolism. The only exception is the genus *Comamonas*, in which steroid catabolism genes were found in four of four genomes.

### Identification of steroid catabolism genes.

Genomes of putative steroid-degrading organisms were subsequently searched by best reciprocal BLASTp analysis to more comprehensively identify steroid catabolism genes and match them to their orthologs among the reference genes. A total of 124 complete genomes were analyzed by BLASTp (see Table S2 in the supplemental material). These included the genomes of all species within each genus identified by HMM analysis. Where species were represented by multiple genomes, a single representative strain was analyzed by BLASTp. The only exception was *Pseudomonas putida*, for which genomes of all strains were analyzed by BLASTp, because only a subset of strains were identified by the HMM analysis. In addition, 75 draft genomes were analyzed by BLASTp (Table S2). These included the genomes of a single strain from each species identified by HMM analysis. Draft *Mycobacterium* genomes were not analyzed, as this genus was very well represented by complete genomes. We additionally conducted BLASTp analysis of 24 *Rhodococcus fascians* genomes. Actinobacterial genomes were queried using 114 protein sequences deduced from the *R. jostii* cholate and cholesterol degradation gene clusters (see Fig. S1), while proteobacterial genomes were queried using 93 protein sequences deduced from the *C. testosteroni* CNB-2 cholate and testosterone degradation gene cluster. The phylogeny of the bacteria was assessed using 16S rRNA gene sequences. Nearly all complete genomes and draft genomes identified by the previous HMM analysis had reciprocal hits to a large majority of query sequences from at least one gene cluster. The only two exceptions among complete genomes were those of *Novosphingobium pentaromativorans* and *Arthrobacter gangotriensis* (not shown). None of the 24 *R. fascians* draft genomes were found to encode a steroid catabolism pathway (not shown).

**FIG 2:**
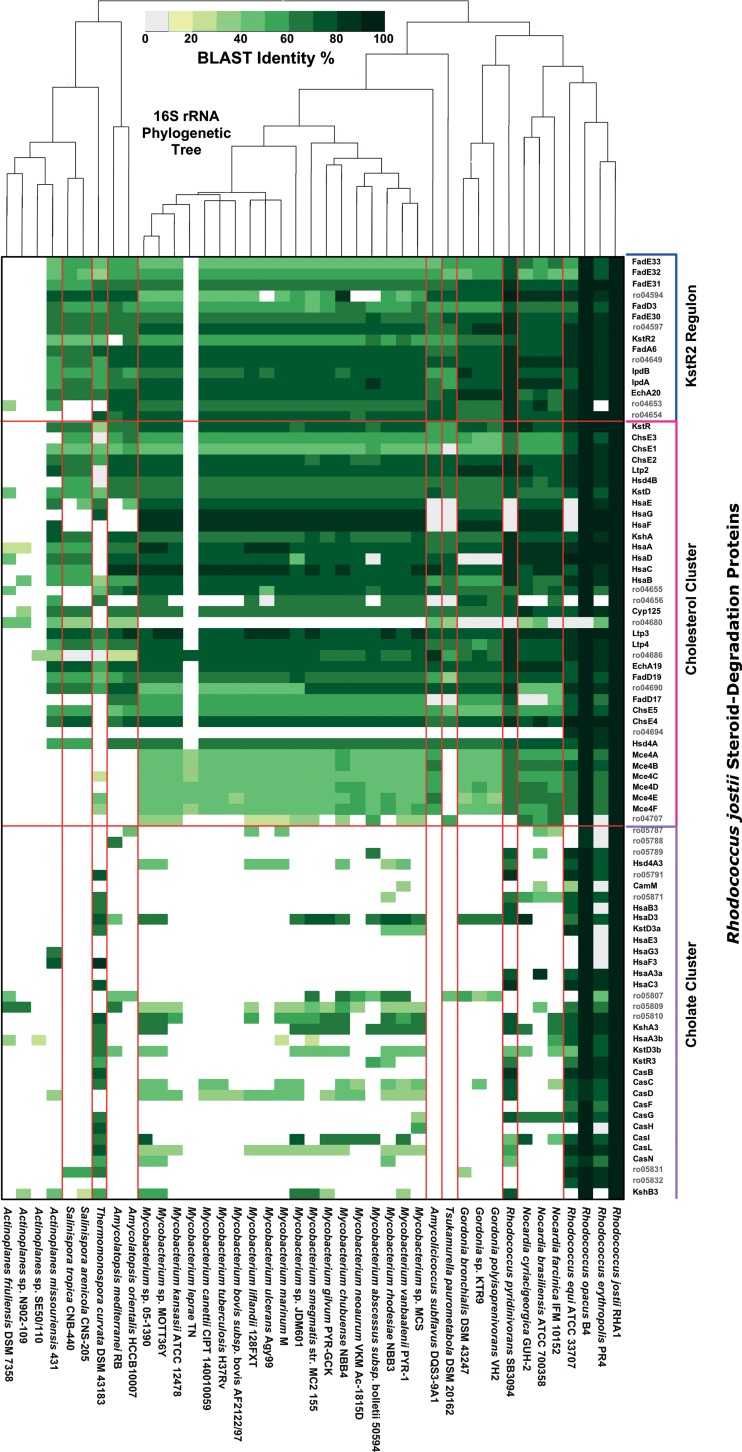
Heat map showing BLAST identity for best reciprocal BLASTp hits to *Rhodococcus jostii* RHA1 steroid degradation proteins in 41 actinobacterial complete genomes.

In all *Actinobacteria* confirmed to have steroid catabolism genes via BLASTp analysis, the hits included orthologs of cholesterol catabolism genes from *R. jostii* (Fig. 2; see also Fig. S3 in the supplemental material). The only exception to this was *Thermomonospora curvata*. The distribution of the actinobacterial cholate pathway was much more restricted than that of the cholesterol pathway, as it was identified only in genomes of *Rhodococcus* spp., *T. curvata*, *Gordonia rubripertincta*, and *Saccharomonospora paurometabolica*.

**FIG 3:**
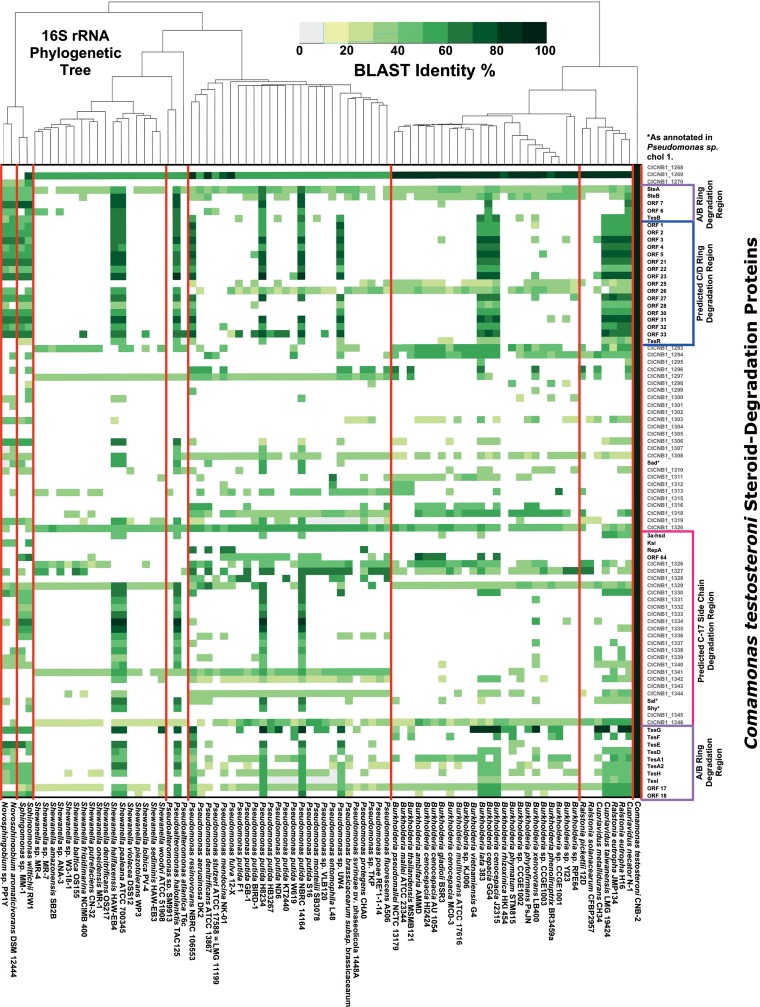
Heat map showing BLAST identity for best reciprocal BLASTp hits to *Comamonas testosteroni* CNB-2 steroid degradation proteins in 82 proteobacterial complete genomes.

In all *Proteobacteria* spp. confirmed to have steroid catabolism genes via BLASTp analysis, the hits included orthologs of testosterone/cholate catabolism genes from strain CNB-2 (Fig. 3; see also Fig. S3 in the supplemental material). In contrast to actinobacterial genera, proteobacterial genera represented by multiple genomes had a minority of members with putative steroid catabolism pathways. And, within the species *Pseudomonas putida*, some strains were predicted to have a steroid catabolism pathway, whereas others were not. Of the *Proteobacteria* with steroid catabolism pathways, all members of *Burkholderia*, *Ralstonia*, *Cupriavidus*, and *Novosphingobium* as well as two of four members of *Pseudomonas* lacked most of the genes associated with degradation of the cholate side chain. Further, the two *Sphingomonas* strains had only a subset of the side chain degradation genes.

### Gene localization.

**FIG 4:**
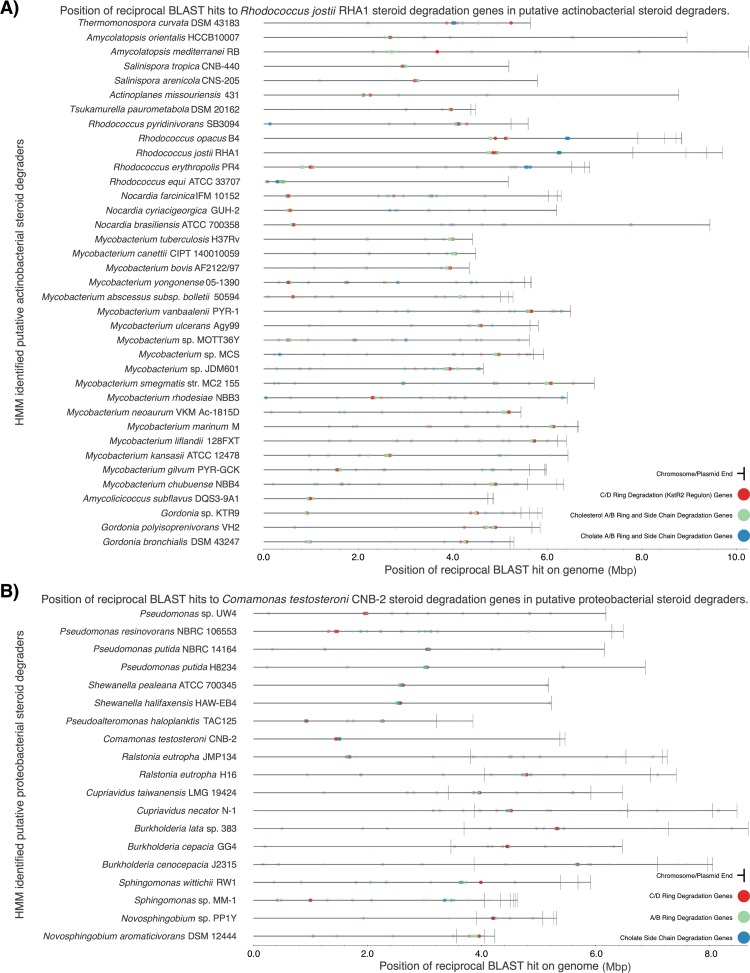
Localization of steroid catabolism genes in genomes. The genes and their functional groupings are as described for Fig. 2 and 3. Each gene is mapped by a translucent dot, so areas of intense color indicate clusters with many genes. (A) *Rhodococcus jostii* RHA1. (B) *Comamonas testosteroni* CNB-2.

The positions in each genome of the BLASTp hits described above were mapped, and there was a strong tendency, as in the reference genomes, for genes to reside in one or a few clusters. Most of the actinobacterial genomes had a single cluster containing most of the cholesterol catabolism genes (Fig. 4A), including genes encoding C/D ring degradation, which are organized in a distinct regulon regulated by KstR2, a Tet-like repressor, in *R. jostii* and *M. tuberculosis* H37Rv. *Rhodococcus* spp. additionally had the cholate catabolism genes in a separate cluster, remote from the cholesterol catabolism cluster and lacking C/D ring degradation genes. *R. equi* was an exception, having a single gene cluster with predicted cholate and cholesterol catabolism genes. There were several other actinobacterial genomes with distinct clustering patterns. *Thermomonospora curvata* had a cluster with cholate degradation genes and a separate one with C/D ring degradation genes. *Mycobacterium abscessus* subsp. *bolletii* had two gene clusters, both with cholesterol catabolism genes. Finally, in *Amycolatopsis mediterranei*, the gene cluster with C/D ring degradation genes was distant from the one encoding the remainder of the cholesterol pathway. Most of the proteobacterial genomes had a single cluster with all of predicted testosterone/cholate catabolism genes (Fig. 4B). Exceptions were *Sphingomonas wittichii* and *Pseudoalteromonas haloplanktis*, in which A/B ring and C/D ring degradation genes are located in separate clusters.

In the vast majority of cases, steroid degradation genes were chromosomally located. One exception is a cluster of genes putatively encoding only A/B ring degradation located on a large linear plasmid, pRHL1, of *R. jostii* (27). These genes are most similar to those encoding the cholate pathway, but their function, if any, is unknown (8). Another exception is a gene cluster putatively encoding the testosterone/cholate pathway in two *Novosphingobium* spp. located on plasmids. Steroid degradation genes were not found on the chromosomes of these two strains.

### Phylogeny of steroid degradation genes.

**FIG 5:**
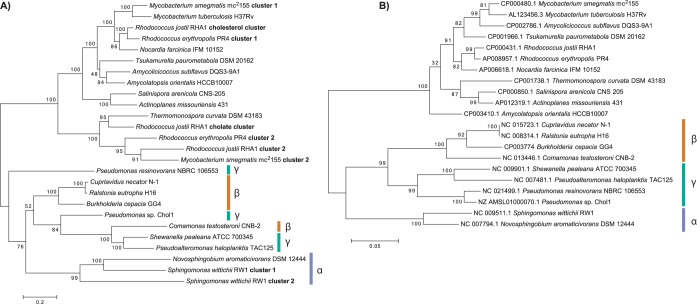
Phylogeny of key steroid degradation enzymes and the corresponding organisms. Bootstrap values are given as percentages of 2,500 repetitions. (A) Phylogeny of orthologs of KshA/CtCNB1_1306, HsaA/TesA1, HsaC/TesB, and HsaD/TesD. The dendrogram is based on concatenated sequences of the four proteins from each steroid catabolism gene cluster found in each bacterium. The scale corresponds to 0.2 substitutions per amino acid. (B) Phylogeny of the 16S rRNA genes of each organism. The scale corresponds to 0.05 substitutions per nucleotide.

The phylogeny of a subset of four key steroid degradation enzymes, KshA/CtCNB1_1306, HsaA/TesA1, HsaC/TesB, and HsaD/TesD, plus a set of their orthologs was investigated by multilocus sequence analysis. The phylogeny of the proteins reveals two distinct clades for *Actinobacteria* and *Proteobacteria* (Fig. 5). The actinobacterial proteins form subclades corresponding to the steroid substrate (cholesterol or cholate). A third subclade includes proteins of unknown function encoded in gene cluster 2 of *R. jostii* plus proteins from *R. erythropolis* and *M. smegmatis*. The phylogeny of the actinobacterial proteins is congruent with that of the corresponding 16S rRNA genes. In contrast, the phylogeny of the proteobacterial proteins is not congruent with that of the corresponding 16S rRNA genes. Further, the proteins form subclades that include proteins from both beta- and gammaproteobacteria. Only the proteins from alphaproteobacteria form a coherent subclade, which includes proteins encoded in two separate gene clusters found in *Sphingomonas wittichii*.

### Growth on steroids.

To verify our predictions of steroid degradation capacity, we tested nine putative steroid-degrading bacteria, newly identified by the analyses described above, for their ability to grow on or otherwise metabolize cholesterol, cholate, and testosterone. These phylogenetically diverse strains were isolated from a range of environments. Growth was determined as an increase in protein levels attributable to the steroid substrate, and metabolism was confirmed by removal of steroids from the medium or transformation to metabolites. In some cases additional organic substrates were required in the medium, and in some cases tyloxapol or methyl-β-cyclodextrin was required to make steroids bioavailable.

As predicted, all nine strains were able to grow on, or metabolize, at least one of the steroid substrates (Table 2; see also Fig. S4 in the supplemental material). The three nonmarine proteobacterial strains all grew on testosterone, while the marine proteobacterium *Shewanella pealeana*, with the cluster of genes predicted to encode cholate side chain degradation (Fig. 3), metabolized both testosterone and cholate. None of the *Proteobacteria* spp. metabolized cholesterol under any conditions tested.

**TABLE 2:** Growth on or metabolism of three steroids by nine predicted steroid degraders^a^

Phylum	Strain	Steroid substrate		
		Cholesterol	Cholate	Testosterone
*Proteobacteria*	*Pseudomonas resinovorans* NRBC106553	–	–	G, R
	*Cupriavidus necator* ATCC 17699	–	–	G, R
	*Sphingomonas wittichii* RW1	–	–	G, R
	*Shewanella pealeana* ATCC 700345	–	R, a	T, a
*Actinobacteria*	*Thermomonospora curvata* ATCC 19995	–	G, R	T
	*Actinoplanes missouriensis* 431	G, R, t	–	T
	*Salinispora arenicola* CNS-205	R, a, c/t	–	T
	*Amycolicicoccus subflavus* DQS3-9A1T	R, a, c	–	–
	*Amycolatopsis* sp. strain ATCC 39116	G, R	R, a	G, R, c

a Symbols: −, no growth or metabolism; G, growth on steroid as sole organic substrate; T, transformation of steroid to metabolites; R, complete removal of steroid with no detectable metabolite accumulation; t, tyloxapol required; c, methyl-β-cyclodextrin required; c/t, either tyloxapol or cyclodextrin required; a, metabolism in the presence of an additional organic substrate.

Substrate use by the five tested actinobacterial strains was more multifarious, but all strains grew on, or metabolized, either cholesterol or cholate (Table 2; see also Fig. S4 in the supplemental material). As predicted, *Actinoplanes missouriensis*, *Salinispora arenicola*, *Amycolicicoccus subflavus*, and *Amycolatopsis* sp. all grew on or otherwise metabolized cholesterol. *Amycolatopsis* sp. additionally grew on testosterone and metabolized cholate. As expected, *Thermomonospora curvata* grew on cholate but not on cholesterol or testosterone.

## DISCUSSION

### Novel steroid degraders.

This study characterized the occurrence of aerobic steroid degradation pathways among more than 8,000 microbes with high-quality genome sequences. We found such pathways only in members of the *Actinobacteria* and *Proteobacteria*, while they do not appear to exist in *Archaea*, fungi, or other bacterial phyla. This taxonomic distribution is consistent with previous studies reporting the enrichment and isolation of microbial steroid degraders (5, 6) and the phylogeny of the known steroid-degrading bacteria. However, within these two phyla, the results of this study substantially expand the range of taxa known to be capable of, or predicted to be capable of, steroid degradation. We provide the first evidence for this capacity in members of the genera *Actinoplanes*, *Aeromicrobium*, *Amycolatopsis*, *Amycolicicoccus*, *Burkholderia*, *Cupriavidus*, *Glaciecola*, *Hydrocarboniphaga*, *Marinobacterium*, *Nocardia*, *Nocardioides*, *Ralstonia*, *Saccharomonospora*, *Salinispora*, *Shewanella*, *Streptomyces*, and *Thermomonospora*. Our growth experiments confirmed predictions of steroid metabolism by *Actinoplanes*, *Cupriavidus*, *Salinispora*, *Shewanella*, *Thermomonospora*, and *Amycolicicoccus* (Table 2; see also Fig. S4 in the supplemental material). As discussed below, our growth experiments also generally confirmed predictions of substrate specificities of the pathways.

A caveat of this analysis is that it cannot identify steroid degradation pathways that are nonhomologous to, or extremely divergent from, the reference pathways. Furthermore, microbial taxa are not equally represented by RefSeq genome sequences, so the probability of identifying steroid degradation pathways in poorly represented taxa was lower. Since genome sequences were not available for the denitrifying *Proteobacteria* spp. mentioned above that anaerobically degrade cholesterol and testosterone, we could not include them in our analysis. Thus, additional pathways may remain to be discovered.

### Distribution of pathways among taxa.

The cholesterol pathway genes are part of a core genome shared by members of most genera within the suborder *Corynebacterineae* (Fig. 2; see also Fig. S3 in the supplemental material). Accordingly, some members of *Dietzia*, *Mycobacterium*, *Gordonia*, *Rhodococcus*, and *Tsukamurella* were previously shown to degrade cholesterol and use it as a growth substrate (5, 6, 9, 28). The cholesterol pathway was found in additional suborders within the *Actinobacteria*, but its distribution there is generally unclear due to the limited number of genome sequences representing most of these taxa. The cholesterol pathway occurs but is not conserved in some actinobacterial genera, as it was found in only 1 in 4 *Actinoplanes* spp., 1 in 6 *Saccharomonospora* spp., and 1 in over 50 *Streptomyces* spp*.*

Additionally, the cholate pathway genes are part of the core genome of the genus *Rhodococcus*. Interestingly, although *Rhodococcus* spp. do not appear to be monophyletic (29), the cholate pathway is conserved among *Rhodococcus* spp. and not among members of closely related genera, such as *Nocardia* and *Gordonia* (Fig. 2). *Rhodococcus fascians* is an exception, as it lacks both the cholesterol and cholate pathways. *R. fascians* comprises plant pathogens and other plant-associated strains (30), which presumably would not benefit from the ability to catabolize the two animal steroids and therefore have lost the corresponding genes. The cholate pathway genes may help to resolve the complex taxonomy of *Rhodococcus* and related genera.

In contrast to the distribution of steroid degradation pathways in *Actinobacteria*, the distribution of the testosterone/cholate pathway among proteobacterial taxa is generally patchy. Thus, in proteobacterial genera represented by multiple genome sequences, we found the testosterone/cholate pathway genes in only one or a few of those genomes (Fig. 3; see also Fig. S3 in the supplemental material). And, among strains of *Pseudomonas putida*, we found those genes in only a few of many strains. However, exceptions to this trend are the genus *Comamonas*, in which all four species with sequenced genomes have the pathway, and the SAR86 cluster, in which all three strains with sequenced draft genomes have the pathway. Recently, genotypic analysis of 14 *Comamonas testosteroni* strains revealed that the testosterone/cholate degradation pathway is part of the core genome of this species (31). Unfortunately, most of these genomes were not available at the time of our analysis.

### Specificity of pathways.

Culture-based experiments were largely consistent with the bioinformatic predictions of the abilities of strains to metabolize particular steroids. Thus, the three nonmarine proteobacterial strains tested consistently grew on testosterone, while *Shewanella pealeana*, with genes encoding cholate side chain degradation, metabolized both testosterone and cholate. The inability of these *Proteobacteria* spp. to metabolize cholesterol is in agreement with our prediction and previous reports of steroid-degrading *Proteobacteria* unable to degrade sterols, such as cholesterol, with alkyl side chains (4, 5). In *Proteobacteria* spp., the inability to degrade cholesterol is consistent with the absence of orthologs of the P450 monooxygenases Cyp125 and Cyp142. These enzymes are used by *Actinobacteria* to oxidize steroid alkyl side chains to initiate their degradation (15–17, 32, 33). In the *Proteobacteria* spp. that have been examined, degradation of the steroid side chain is a prerequisite for subsequent steroid nucleus degradation (34, 35). The lack of a transporter, such as the Mce4 system, may also contribute to the inability of *Proteobacteria* to metabolize cholesterol. Overall, there is currently no evidence suggesting that members of the *Proteobacteria* can catabolize cholesterol via the 9,10-seco pathway.

As predicted, four actinobacterial strains with cholesterol degradation gene clusters all metabolized cholesterol. *Amycolatopsis* sp. additionally grew with testosterone, suggesting that the pathway for cholesterol catabolism can also support catabolism of testosterone in some bacteria, an ability that was not previously recognized. *Salinispora arenicola* and *Amycolicicoccus subflavus* completely removed cholesterol from their medium but failed to grow. This unexpected result may be related to the fact that they are very slow growing, even on rich LB medium. The ability of *Amycolatopsis* sp. to metabolize cholate despite its lacking an actinobacterial cholate degradation gene cluster indicates that the actinobacterial cholesterol degradation cluster can also support degradation of cholate for some organisms.

As predicted, *Thermomonospora curvata* grew on cholate but not on cholesterol. It also metabolized testosterone, indicating that the actinobacterial cholate degradation pathway also has this capacity. This strain offers a rare opportunity to examine the actinobacterial cholate degradation pathway in isolation, verifying that it is sufficient for catabolism of cholate. Most actinobacterial genomes encoding the cholate pathway also encode the cholesterol pathway, and some *Rhodococcus* spp. have further clusters of steroid degradation genes of unknown function (8, 9). Overall, our culture-based experiments add credibility to the bioinformatic predictions of steroid catabolism by *Proteobacteria* and *Actinobacteria*. Further supporting our predictions, *Gordonia* sp. strain KTR9, predicted to have a cholesterol degradation cluster, was previously shown to grow with cholesterol but not with cholate or testosterone (36).

### Evolution of pathways.

The distributions of steroid degradation pathways among taxa suggest a possible scenario for evolution and dissemination of the pathways. This scenario involves evolution of a single ancestral pathway, since the known aerobic pathways are all homologous. The most parsimonious interpretation of our results is that the pathway originated in an ancestor of the *Corynebacterineae* and gave rise to the cholesterol pathway. This ancestry is consistent with our findings, including the nearly ubiquitous occurrence of the pathway in most genera within this suborder. However, this ancestry is speculative, and even the possibility of an origin in the *Proteobacteria* cannot be excluded. A more comprehensive phylogenetic analysis, and perhaps more genome sequences, would be required to better establish ancestry.

The distribution of the actinobacterial cholate pathway suggests that it originated via a duplication of the cholesterol pathway genes in an ancestor of *Rhodococcus*. The presence of more than two clusters of homologous steroid degradation genes in several *Rhodococcus* spp. suggests multiple duplications of these genes. Genes encoding steroid A/B ring degradation are found in gene clusters for both the cholesterol and cholate pathways, while, notably, the cluster of genes encoding steroid C/D ring degradation did not duplicate and is found linked only to the cholesterol pathway genes. The occurrence of the cholesterol or cholate pathway in *Actinobacteria* beyond the *Corynebacterineae* could be due to either vertical or horizontal transmission, as the limited availability of genome sequences representing these taxa does not strongly support either possibility. The patchy distribution of the monophyletic testosterone/cholate pathway among *Proteobacteria* spp. suggests a single horizontal transfer of an actinobacterial steroid pathway to a proteobacterium, followed by horizontal distribution among *Proteobacteria*. The concept of horizontal transfer among *Proteobacteria* is further supported by the phylogeny of four key proteins in the three reference pathways, which is not congruent with the phylogeny of the corresponding 16S rRNA genes (Fig. 5). The observed clustering of steroid degradation genes within genomes (Fig. 4) and the location of some clusters on plasmids both likely facilitated horizontal transfer of entire pathways or major components of pathways. In particular, a gene cluster with greatest similarity to that of the actinobacterial cholate pathway genes located on plasmid pRHL1 in *R. jostii* may have facilitated horizontal transfer of that pathway, although the function of those genes in *R. jostii* is unclear. It is noteworthy that such large, linear, single-copy plasmids may exist unrecognized in draft or incompletely assembled genomes. The location of testosterone/cholate pathway genes on plasmids in *Novosphingobium* spp. is consistent with horizontal transfer of the pathway to this genus. While the evolutionary scenario described above is most parsimonious with respect to the available evidence, it remains speculative.

### Ecology.

The majority of putatively steroid-degrading bacteria that we identified were isolated from soil, host, and aquatic environments (see Fig. S2 in the supplemental material). Our analysis particularly extends knowledge of steroid-degrading marine bacteria, including members of *Glaciecola*, *Marinobacterium*, *Pseudoalteromonas*, *Shewanella*, OM60, and SAR86, which originated from marine environments (37–42). Although some *Vibrio* species within the gammaproteobacteria, without sequenced genomes, have been characterized as marine steroid degraders (43, 44), we did not find steroid degradation genes in 186 genomes of marine *Vibrio* spp. that we analyzed, which is consistent with the patchy distribution of steroid degradation pathways among proteobacterial taxa.

In soil and aquatic environments, steroids constitute a significant resource for heterotrophic bacteria, and steroid degraders function in decomposition of eukaryotic biomass and excreta from vertebrates. Accordingly, many taxa identified in this study are associated with biomass decomposition. Notably, the *R. jostii* type strain was isolated from the sarcophagus of a medieval knight (45), so it is tempting to speculate that *R. jostii* participated in degrading the corpse and then survived centuries of dormancy. The strong conservation of the genes encoding steroid degradation pathways in core genomes of actinobacterial taxa indicates that this catabolism is fundamental to their life history. Thus, cholesterol catabolism appears important to niches of members of several actinobacterial genera, including most in the *Corynebacterineae*, while cholate catabolism additionally appears to be important to the niches of most members of *Rhodococcus*. In contrast, the patchy distribution of the testosterone/cholate pathway among members of *Proteobacteria* indicates that individual, distantly related proteobacterial species or strains have adopted niches involving steroid catabolism.

In addition to free-living species, a substantial proportion of the cholesterol-degrading *Actinobacteria* that we identified, including members of *Mycobacterium*, *Rhodococcus*, and *Nocardia*, are pathogens of mammals (46–48). These pathogenic species tend to have reduced genomes, but within these genera, only the obligately intracellular pathogen *Mycobacterium leprae* has lost the steroid degradation genes. Recent studies showed that *M. leprae* has indeed lost the ability to metabolize cholesterol as a carbon and energy source (49) but still requires host cholesterol for intracellular survival (50). Conservation of the pathway in all other pathogenic species in those genera suggests that cholesterol catabolism is important to their niches. Indeed, several lines of evidence indicate that catabolism of host cholesterol is essential for *M. tuberculosis* survival *in vivo* (9, 13, 51) and for pathogenesis of *R. equi*, which infects foals (52).

### Further delineation of steroid degradation genes.

Our comparisons of a large number of steroid catabolism gene clusters shed new light on the involvement of particular genes in the respective pathways. Uncharacterized genes *ro04680* and *ro04694* from the *R. jostii* cholesterol pathway gene cluster are not conserved in other putative cholesterol degraders and so are unlikely to be associated with the pathway. The *mce4* genes, encoding a cholesterol/sitosterol uptake system (18), are conserved only in mycolic acid bacteria within the *Corynebacterineae*. The only complete genome from a mycolic acid bacterium with the cholesterol pathway but lacking the *mce4* genes is that of *Tsukamurella paurometabola*. The Mce4 uptake system may be specifically adapted for transport across the distinct cell envelope of mycolic acid bacteria, which includes a true outer membrane (53). Steroid uptake systems in other taxa or systems for other steroid substrates have not been identified.

The restricted distribution of the actinobacterial cholate pathway limits the comparison of genomes to verify genes associated with the pathway, but some conclusions are possible. The lack of conservation of *ro05807* and *ro05809* supports the previous conclusion, based on transcriptomic analysis, that these genes are not associated with the pathway (8). Similarly, *camM* is not conserved, in accordance with the previous study showing that CamM is a transporter functioning in reassimilation of a cholate metabolite by *R. jostii* which is not essential for growth on cholate (54). It also appears that the *casH* gene may not be essential to the pathway.

A single large cluster of genes in the genomes of *C. testosteroni* strains TA441 and CNB-2 encodes the proteobacterial testosterone/cholate pathway (see Fig. S1 in the supplemental material). Our analysis suggests that a subset of 26 genes in this cluster (*C. testosteroni* CNB-2 *CtCNB1_1293* through *CtCNB1_1320*) is not associated with the pathway. An exception within that subset is *CtCNB1_1306*, which is conserved in most genomes encoding the pathway but not in genomes of related organisms that do not encode the pathway. The product of this gene is associated via BLASTp clustering with actinobacterial KshA, the large subunit of 3-ketosteroid-9α-hydroxylase (KSH), which cleaves steroid B ring (55). The putative role of this gene in *C. testosteroni* and other *Proteobacteria* was not previously recognized. However, the *ORF17* gene in strain CNB-2 was predicted to encode KshB, the small subunit of KSH (4), a conclusion which is further supported by our analysis.

We further predict that genes *CtCNB1_1308*, *CtCNB1_1309*, *CtCNB1_1310*, *ORF6*, *ORF7*, *CtCNB1_1347*, and *CtCNB1_1348* in strain CNB-2 are involved in cholate degradation but not in testosterone degradation, as they are conserved in *Proteobacteria* predicted to degrade both substrates but not in those predicted to degrade only testosterone. Accordingly, *CtCNB1_1309*, *CtCNB1_1347*, and *CtCNB1_1348* are homologs of the cholate side chain degradation genes *sad*, *sal*, and *shy*, respectively, which were identified in *Pseudomonas* sp. strain Chol1 (11). Similarly, *ORF6* and *ORF7* are homologs of the genes *sor1* and *hsh1*, respectively, which were identified in strain Chol1 and are required for the degradation of steroids with a hydroxyl group at C12 (56). Our analysis suggests that these genes are not associated with testosterone degradation but only with cholate degradation. Nevertheless, Horinouchi et al. have shown that transcription of *ORF6* is induced during growth with both substrates (35). In addition, our analysis supports the previous prediction that *CtCNB1_1330* to *CtCNB1_1340* encode the remaining steps of cholate side chain degradation (4, 57), because these genes are conserved only in organisms predicted to degrade cholate. Furthermore, *CtCNB1_1308* encodes a putative MFS transporter, which could be involved in uptake of cholate or one of its degradation intermediates. Among the cluster of genes in strain CNB-2 predicted to encode C/D ring degradation (4), our analysis supports the previously published notion (31) that the *ORF25* and *ORF26* genes are not associated with the testosterone/cholate pathway.

Finally, homologs of genes *hsaE* and *tesE*, genes *hsaF* and *tesG*, and genes *hsaG* and *tesF* are not conserved in many of the steroid catabolism gene clusters. These genes encode a series of reactions that are common to many catabolic pathways that involve meta-cleavage of aromatic rings (58, 59). Thus, it appears that many steroid degraders have genes elsewhere in their genomes encoding enzymes catalyzing one or more of these three reactions. Accordingly, *hsaE*, *hsaF*, and *hsaG* from the cholesterol degradation gene cluster of *M. tuberculosis* strain H37Rv have been shown to be nonessential for growth on cholesterol (60).

## MATERIALS AND METHODS

Bioinformatic software environments and packages as well as growth experiments used in this study are described in the supplemental Methods (see Text S1 in the supplemental material).

### Steroid catabolism reference proteins.

Predicted proteins encoded by previously characterized steroid catabolism genes from three bacterial strains served as initial reference proteins. These strains were *Rhodococcus jostii* RHA1 (RefSeq NC_008268.1), *Mycobacterium tuberculosis* H37Rv (RefSeq NC_000962.3), and *Comamonas testosteroni* CNB-2 (RefSeq NC_013446.2). Strain CNB-2 was the only *C. testosteroni* strain with a complete genome in NCBI’s GenBank at the time of this investigation, and it was selected due to the fact that the sequenced and characterized steroid degradation genes of *C. testosteroni* TA441 were mapped to the strain CNB-2 genome in a recent publication (4).

The initial reference proteins were binned into homologous groups using BLASTp (61) (v2.2.29; http://blast.ncbi.nlm.nih.gov/Blast.cgi?PAGE=Proteins), applying a minimum identity filter of 30% and a maximum *E* value filter of 10^−30^. The latter two parameters were selected empirically in order to yield clear clusters within the network, which shared common GenBank annotations. Additional reference genes were obtained from the genomes of organisms related to those three strains. On 6 February 2014, 105 genomes (see Table S3 in the supplemental material) were downloaded from NCBI’s GenBank, comprising all draft and complete genomes from *Rhodococcus*, *Mycobacterium*, and *Comamonas* spp. plus a subset from *Streptomyces* spp. A custom program, BackBLAST (v1.0, Lee Bergstrand; https://github.com/LeeBergstrand/BackBLAST_Reciprocal_BLAST), was used to search the predicted proteins of these genomes for putative orthologs (reciprocal BLASTp hits) of the initial reference proteins. A maximum *E* value of 10^−30^ and a minimum identity of 25% were used to maximize stringency while not removing annotated orthologs of the reference proteins, and all other BLASTp settings were default values.

### Generation of HMMs.

Eight proteins were selected for HMM development (Table 1), because (i) they unambiguously clustered in orthologous groups, (ii) they occur in all three reference pathways, and (iii) they have known functions in steroid nucleus degradation. Each of these proteins from *R. jostii*, *M. tuberculosis*, and *C. testosteroni* plus their reciprocal BLASTp hits were subclustered with CD-hit (62) (v4.6.1; https://github.com/weizhongli/cdhit), using a minimum sequence identity of 50%, a word size value of 3, and all other parameters left at default, yielding between 1 and 5 smaller subclusters per input protein. Typically, these subclusters represented proteins of similar taxonomic origins and/or substrate specificities. As an additional filtering step, potentially nonorthologous proteins that did not cluster with the initial eight proteins were removed. Sequences from each of the resulting 25 subclusters were aligned and manually trimmed using Mega (63) (v5.2.2; http://www.megasoftware.net/). The sets of aligned protein sequences were used to generate 25 hidden Markov model (HMMs) using HMMER (64) (v3.1b1; http://hmmer.janelia.org). The HMMs developed are available online (https://github.com/MohnLab/Mohn_Lab_Steroid_Degradation_HMM_Analysis_2015).

### HMM searches.

On 15 May 2014, all complete bacterial and archaeal genomes, plus all complete and draft fungal genomes, were downloaded from NCBI’s curated RefSeq database (2,788 genomes). On 30 July 2014, all incomplete bacterial and archaeal genomes were downloaded from RefSeq (5,489 genomes). Annotated proteins from these genomes were searched with a custom program, HMMER-DB (v1.0, Lee Bergstrand; https://github.com/LeeBergstrand/HMMER-DB), which stores HMM hits generated by HMMER’s hmmsearch in a searchable database. A maximum HMMER *E* value was empirically optimized to 10^−25^, which identified previously known steroid catabolism genes while providing maximum stringency against false positives. All proteins of organisms identified as potential steroid degraders by HMM searches were subsequently searched for best reciprocal BLAST hits to initial reference proteins via BackBLAST, filtering for a minimum identity of 25% and maximum *E* value of 10^−25^ in accordance with previous BLAST and HMM criteria. Additionally, 24 *Rhodococcus fascians* genomes were downloaded from GenBank on 12 August 2015 and searched for best reciprocal BLAST hits.

### Phylogenetic analysis.

The protein sequences for KshA/CtCNB1_1306, HsaA/TesA1, HsaC/TesB, and HsaD/TesD from the reference strains and sequences of their orthologs from 18 additional strains, identified by HMM analysis, were used for phylogenetic analysis. Homologous sequences were aligned using the Muscle algorithm (65) from Mega v6.06 and manually trimmed. The resulting four sequences inferred from each gene cluster were concatenated. Phylogenetic reconstruction was performed with the concatenated protein sequences as well as the 16S rRNA gene sequences of the corresponding organisms, using the maximum likelihood model with default parameters and 2,500 bootstrap replications in Mega v6.06.

## SUPPLEMENTAL MATERIAL

Figure S1  Steroid catabolism gene clusters of *Rhodococcus jostii* RHA1, *Mycobacterium tuberculosis* H37Rv, *Comamonas testosteroni* CNB-2, and *Pseudomonas* sp. strain Chol1. Filled arrows indicate characterized genes and proteins, open arrows indicate annotated genes, and gray arrows indicate genes and proteins probably not involved in steroid degradation. Gene names correspond to protein names in Fig. 1 or gene locus tags, which have been abbreviated to the last digits for strains CNB-2 and Chol1. Download Figure S1, PDF file, 0.4 MB

Figure S2  Sources of isolates whose genomes were found to have steroid catabolism genes via HMM analysis. Download Figure S2, PDF file, 0.7 MB

Figure S3  Heat map showing BLAST identity for best reciprocal BLASTp hits to *Rhodococcus jostii* RHA1 and *Comamonas testosteroni* CNB-2 steroid degradation proteins in 75 bacterial draft genomes. Download Figure S3, PDF file, 1.7 MB

Figure S4  Growth on or metabolism of three steroid substrates by nine predicted steroid degraders. (A) Growth of six strains, measured as protein production, on cholate or cholesterol and removal of the steroid substrates. For *A. missouriensis* 431, differential protein yield was calculated by subtracting the protein yield in control cultures without a steroid substrate. (B) Removal of cholesterol or cholate in the presence of additional organic substrates by four strains. The results from two representative replicate cultures (A and B) and a noninoculated control (ni) are shown. Download Figure S4, PDF file, 0.6 MB

Table S1  Table listing 265 putatively steroid-degrading bacteria identified by HMM analysis. Table S1, XLSX file, 0.5 MB

Table S2  Table listing 124 complete and 75 draft genomes of putatively steroid-degrading bacteria that were analyzed by reciprocal BLASTp analysis. Table S2, XLSX file, 0.5 MB

Table S3  Table listing 105 genomes from GenBank of *Rhodococcus*, *Mycobacterium*, *Comamonas*, and *Streptomyces* spp. that were sources of reference genes. Table S3, XLSX file, 0.5 MB

Text S1  Materials and methods used for bioinformatic analysis and growth experiments. Download Text S1, PDF file, 0.1 MB
