# Erratum for Bergstrand et al., Delineation of Steroid-Degrading Microorganisms through Comparative Genomic Analysis

**DOI:** 10.1128/mBio.00865-16

**Published:** 2016-07-26

**Authors:** Lee H. Bergstrand, Erick Cardenas, Johannes Holert, Jonathan D. Van Hamme, William W. Mohn

**Affiliations:** a Department of Microbiology and Immunology, Life Sciences Institute, University of British Columbia, Vancouver, British Columbia, Canada; b Department of Biological Sciences, Thompson Rivers University, Kamloops, British Columbia, Canada

## ERRATUM

Volume 7, no 2, doi:10.1128/mBio.00166-16, 2016. Some chemical structures in the downstream AB- and CD-ring degradation pathways in Fig. 1 were incorrect. The revised Fig. 1 (below) shows the correct structures.

**FIG 1:**
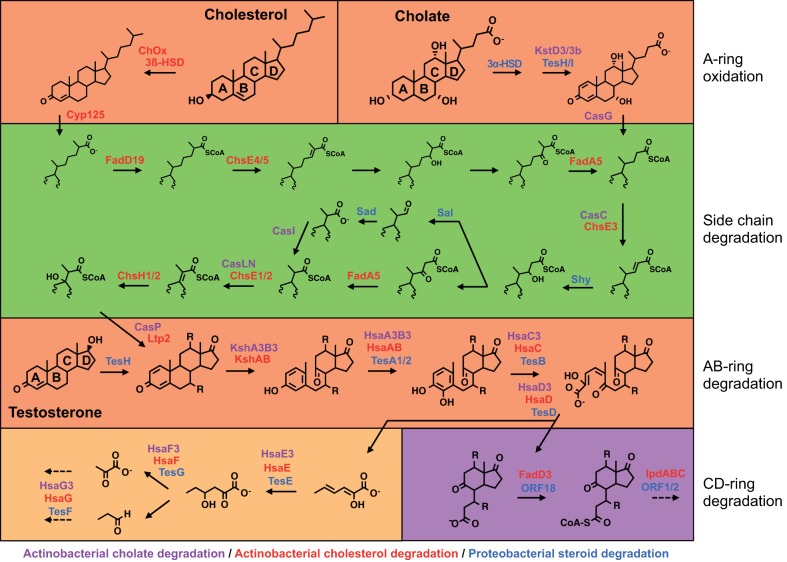
Aerobic 9,10-seco-steroid degradation pathways for cholesterol, cholate, and testosterone. The steroid ring structure is degraded by oxygen-dependent opening and subsequent hydrolytic cleavage of rings A and B. Subsequent degradation of the C and D rings occurs by a mechanism not yet reported. In *Actinobacteria*, side chain degradation and ring opening can occur simultaneously. Characterized or annotated enzymes involved in the degradation of cholesterol by *Actinobacteria* are shown in red, those involved in the degradation of cholate by *Actinobacteria* are shown in lilac, and those involved in the degradation of testosterone or cholate by *Proteobacteria* are shown in blue. Protein nomenclature is based on that for *Rhodococcus jostii* RHA1, *Mycobacterium tuberculosis* H37Rv, *Comamonas testosteroni* TA441, and *Pseudomonas* sp. strain Chol1, and not all proteins are named.

