# Supplementary material for: Delineation of Steroid-Degrading Microorganisms through Comparative Genomic Analysis
Source: mBio. 2016 Mar 8;7(2):e00166-16. doi: 10.1128/mBio.00166-16 (PMC4810484; doi:10.1128/mBio.00166-16)
Supplement: Figure S1 — Steroid catabolism gene clusters of Rhodococcus jostii RHA1, Mycobacterium tuberculosis H37Rv, Comamonas testosteroni CNB-2, and Pseudomonas sp. strain Chol1. Filled arrows indicate characterized genes and proteins, open arrows indicate annotated genes, and gray arrows indicate genes and proteins probably not involved in steroid degradation. Gene names correspond to protein names in Fig. 1 or gene locus tags, which have been abbreviated to the last digits for strains CNB-2 and Chol1. Download [file mbo001162715sf1.pdf]

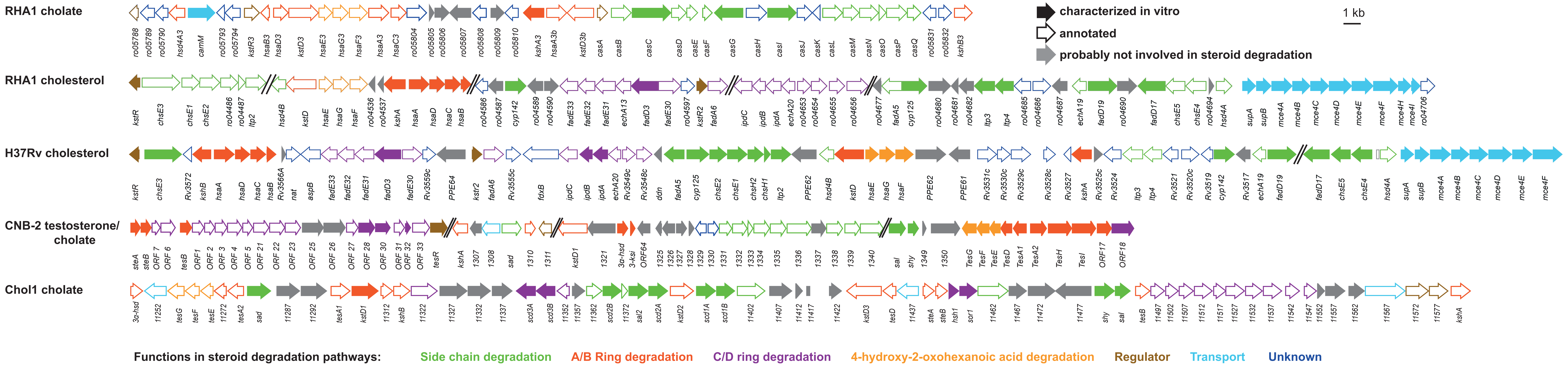

RHA1 cholate
ro05788 ro05789 ro05790 hsd4A3 camM ro05793 ro05794 kstR3 hsaB3 hsaD3 kstD3 hsaE3 hsaG3 hsaF3 hsaA3 hsaC3 ro05804 ro05805 ro05806 ro05807 ro05808 ro05809 ro05810 kshA3 hsaA3b kstD3b casA casB casC casD casE casF casG casH casI casJ casK casL casM casN casO casP casQ ro05831 ro05832 kshB3
characterized in vitro
annotated
probably not involved in steroid degradation
1 kb
RHA1 cholesterol
kstR chsE3 chsE1 chsE2 ro04486 ro04487 ltp2 hsd4B kstD hsaE hsaG hsaF ro04536 ro04537 kshA hsaA hsaD hsaC hsaB ro04586 ro04587 cyp142 ro04589 ro04590 fadE33 fadE32 fadE31 echA13 fadD3 fadE30 ro04597 kstR2 fadA6 ipdC ipdB ipdA echA20 ro04653 ro04654 ro04655 ro04656 ro04677 fadA5 cyp125 ro04680 ro04681 ro04682 ltp3 ltp4 ro04685 ro04686 ro04687 echA19 fadD19 ro04690 fadD17 chsE5 chsE4 ro04694 hsd4A supA supB mce4A mce4B mce4C mce4D mce4E mce4F mce4H mce4I ro04706
H37Rv cholesterol
kstR chsE3 Rv3572 kshB hsaA hsaD hsaC hsaB Rv3566A nat aspB fadE33 fadE32 fadE31 fadD3 fadE30 Rv3559c PPE64 kstr2 fadA6 Rv3555c fdxB ipdC ipdB ipdA echA20 Rv3549c Rv3548c ddn fadA5 cyp125 chsE2 chsE1 chsH2 chsH1 ltp2 PPE62 hsd4B kstD hsaE hsaG hsaF PPE62 PPE61 Rv3531c Rv3530c Rv3529c Rv3528c Rv3527 kshA Rv3525c Rv3524 ltp3 ltp4 Rv3521 Rv3520c Rv3519 cyp142 Rv3517 echA19 fadD19 fadD17 chsE5 chsE4 hsd4A supA supB mce4A mce4B mce4C mce4D mce4E mce4F
CNB-2 testosterone/ cholate
steA steB ORF 7 ORF 6 tesB ORF1 ORF 2 ORF 3 ORF 4 ORF 5 ORF 21 ORF 22 ORF 23 ORF 25 ORF 26 ORF 27 ORF 28 ORF 30 ORF 31 ORF 32 ORF 33 tesR kshA 1307 1308 sad 1310 1311 kstD1 1321 3α-hsd 3-ksi ORF64 1325 1326 1327 1328 1329 1330 1331 1332 1333 1334 1335 1336 1337 1338 1339 1340 sal shy 1349 1350 TesG TesF TesE TesD TesA1 TesA2 TesH TesI ORF17 ORF18
Chol1 cholate
3α-hsd 11252 tesG tesF tesE 11272 tesA2 sad 11287 11292 tesA1 kstD1 11312 kshB 11322 11327 11332 11337 scd3A scd3B 11352 11357 11362 scd2B 11372 sal2 scd2A kstD2 scd1A scd1B 11402 11407 11412 11417 11422 kstD3 tesD 11437 steA steB hsh1 sor1 11462 11467 11472 11477 shy sal tesB 11497 11502 11507 11512 11517 11527 11532 11537 11542 11547 11552 11557 11562 11567 11572 11577 kshA
Functions in steroid degradation pathways:
Side chain degradation
A/B Ring degradation
C/D ring degradation
4-hydroxy-2-oxohexanoic acid degradation
Regulator
Transport
Unknown
