# Supplementary figures and images for: Delineation of Steroid-Degrading Microorganisms through Comparative Genomic Analysis
Source: mBio. 2016 Mar 8;7(2):e00166-16. doi: 10.1128/mBio.00166-16 (PMC4810484; doi:10.1128/mBio.00166-16)

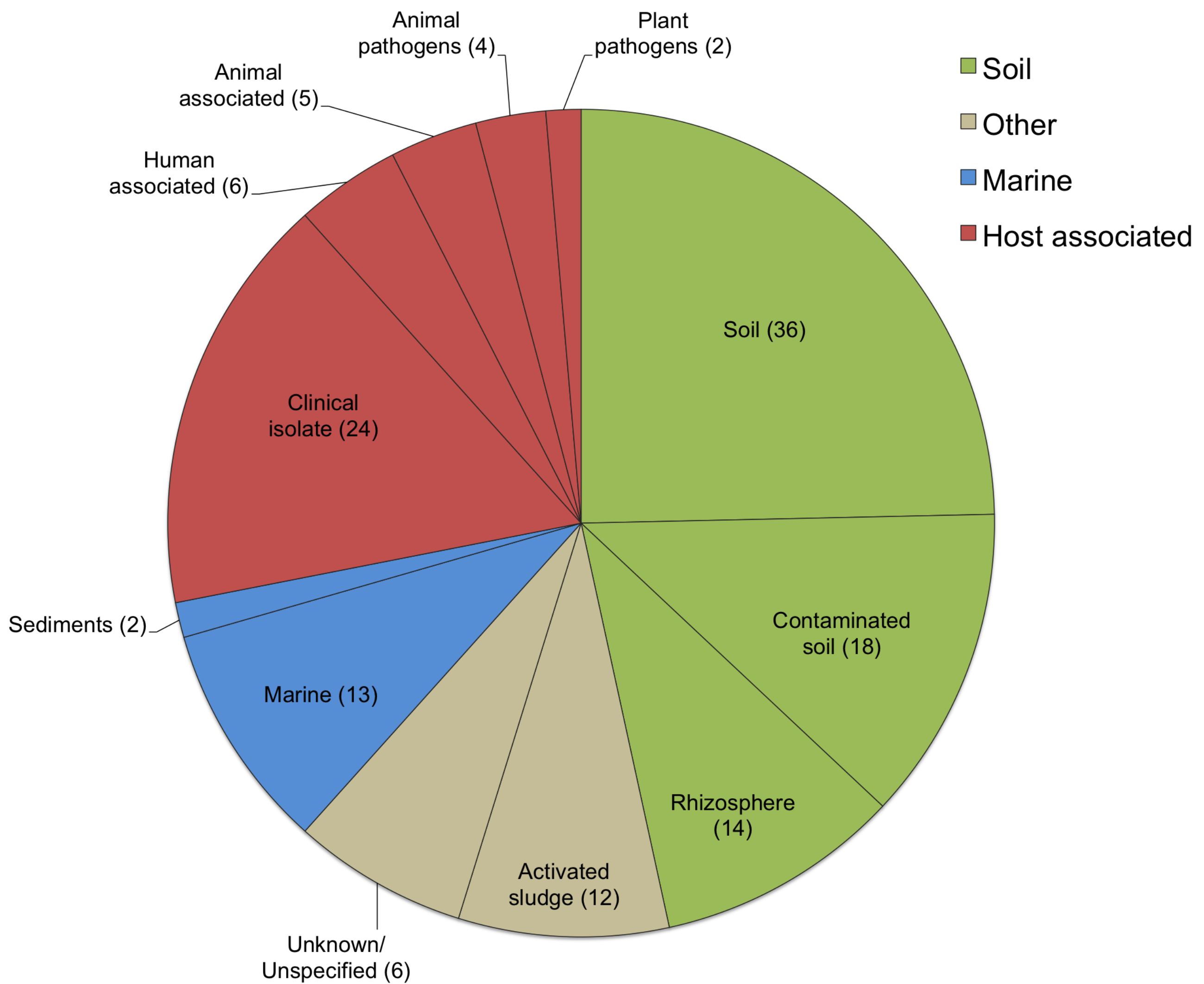

Supplement: Figure S2 — Sources of isolates whose genomes were found to have steroid catabolism genes via HMM analysis. Download [file mbo001162715sf2.pdf]
