# Supplementary material for: Delineation of Steroid-Degrading Microorganisms through Comparative Genomic Analysis
Source: mBio. 2016 Mar 8;7(2):e00166-16. doi: 10.1128/mBio.00166-16 (PMC4810484; doi:10.1128/mBio.00166-16)
Supplement: Figure S3 — Heat map showing BLAST identity for best reciprocal BLASTp hits to Rhodococcus jostii RHA1 and Comamonas testosteroni CNB-2 steroid degradation proteins in 75 bacterial draft genomes. Download [file mbo001162715sf3.pdf]

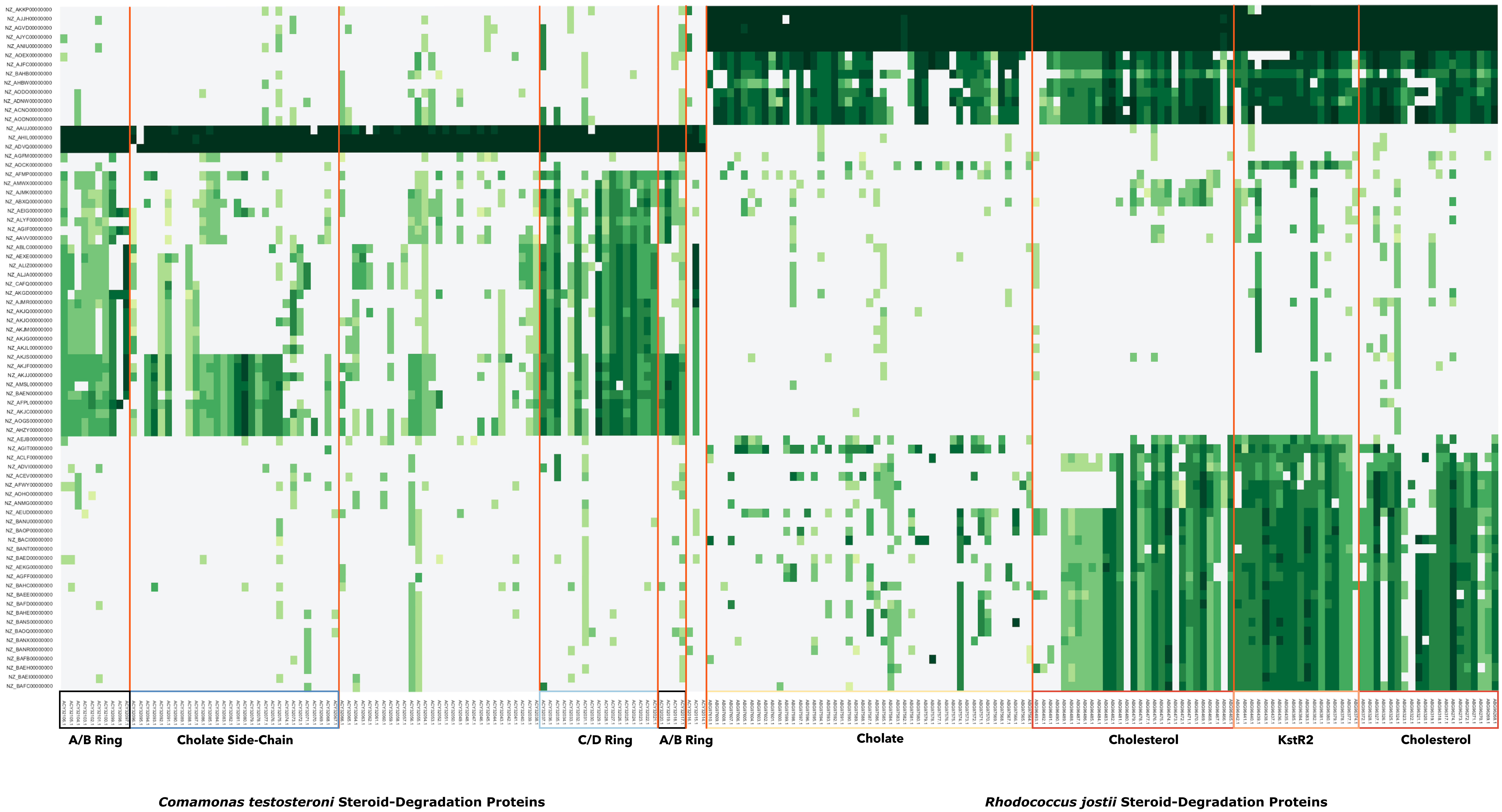
A/B Ring
Cholate Side-Chain
C/D Ring
A/B Ring
Cholate
Cholesterol
KstR2
Cholesterol
Comamonas testosteroni Steroid-Degradation Proteins
Rhodococcus jostii Steroid-Degradation Proteins
