# Supplementary material for: Delineation of Steroid-Degrading Microorganisms through Comparative Genomic Analysis
Source: mBio. 2016 Mar 8;7(2):e00166-16. doi: 10.1128/mBio.00166-16 (PMC4810484; doi:10.1128/mBio.00166-16)

A) Growth of predicted steroid degrading bacteria on steroid substrates

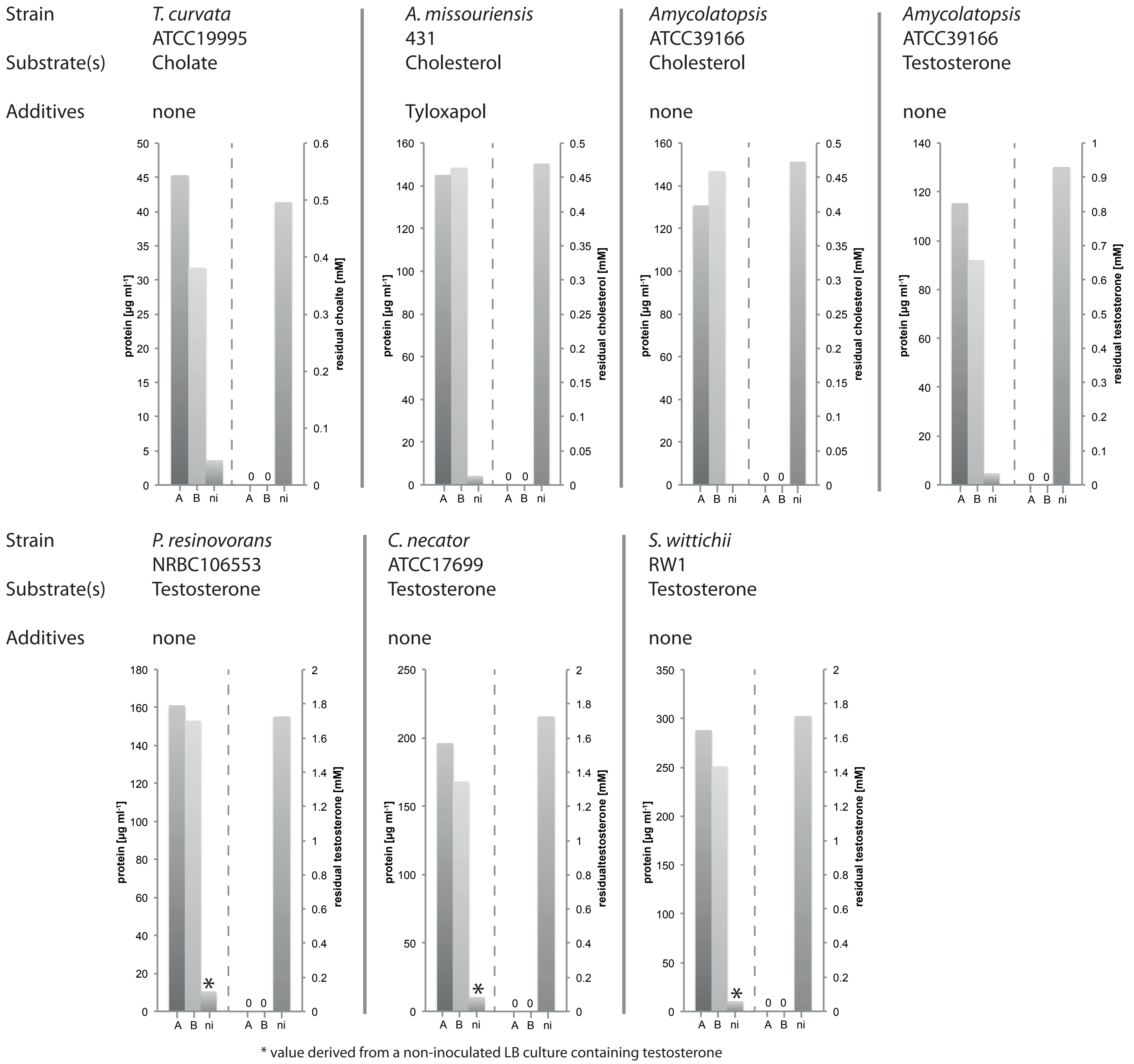

B) Removal of steroid substrates by predicted steroid degrading bacteria

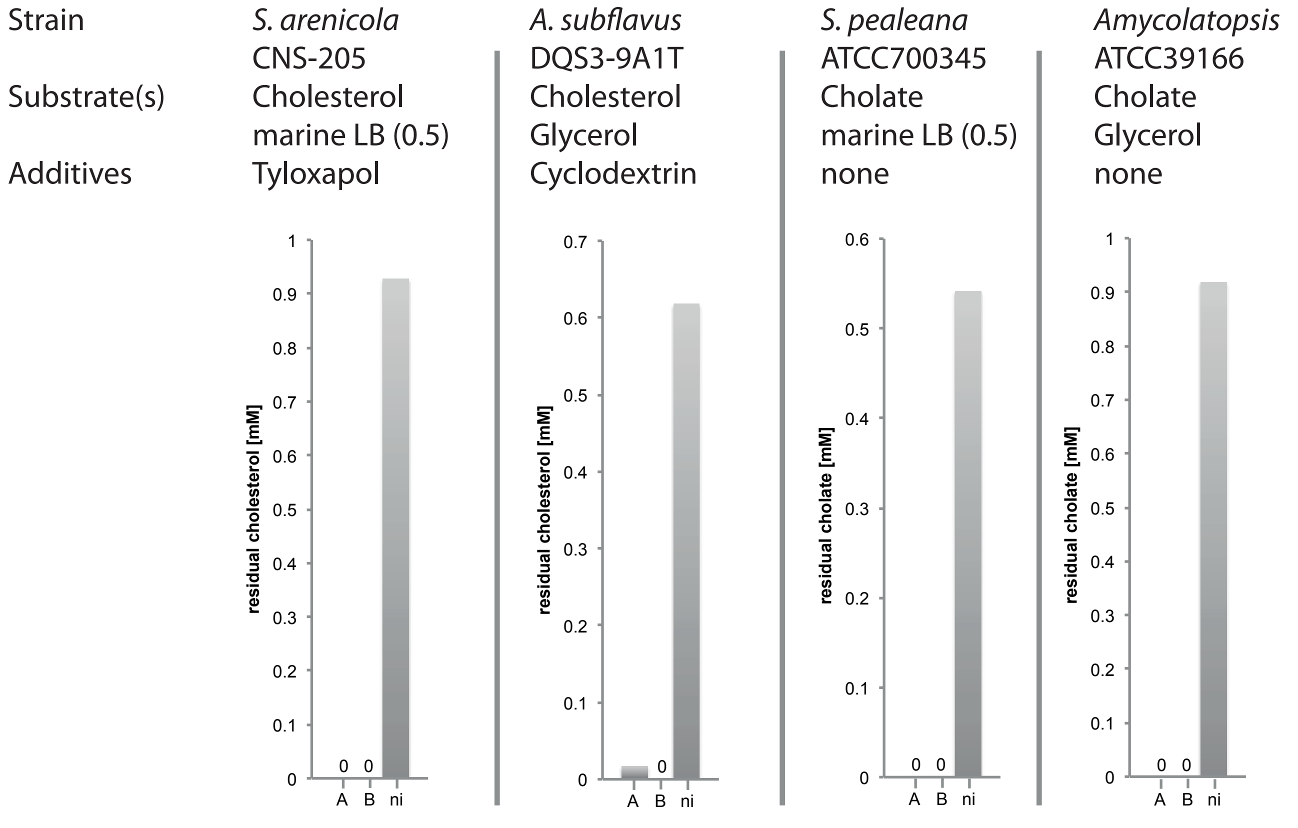

Supplement: Figure S4 — Growth on or metabolism of three steroid substrates by nine predicted steroid degraders. (A) Growth of six strains, measured as protein production, on cholate or cholesterol and removal of the steroid substrates. For A. missouriensis 431, differential protein yield was calculated by subtracting the protein yield in control cultures without a steroid substrate. (B) Removal of cholesterol or cholate in the presence of additional organic substrates by four strains. The results from two representative replicate cultures (A and B) and a noninoculated control (ni) are shown. Download [file mbo001162715sf4.pdf]
