# Supplementary material for: Delineation of Steroid-Degrading Microorganisms through Comparative Genomic Analysis
Source: mBio. 2016 Mar 8;7(2):e00166-16. doi: 10.1128/mBio.00166-16 (PMC4810484; doi:10.1128/mBio.00166-16)
Supplement: Text S1 — Materials and methods used for bioinformatic analysis and growth experiments. Download [file mbo001162715s1.pdf]

Bioinformatics software environments and packages

Data analysis was performed in Python (v2.7.8, Python Software foundation, https://www.python.org), R (v3.1.0, R development core team, https://www.r-project.org) and SQLite (v3.8.4, https://www.sqlite.org). Data was visualized using gplots (v2.11.3, Gregory R. Warnes, https://cran.r-project.org/web/packages/gplots/index.html) and bokeh (v0.9.0, Continuum Analytics, http://bokeh.pydata.org/). In addition, the Python packages pandas (v0.16.2) and numpy (v1.9.2) and the R packages ape (v3.1), reshape2 (v1.4) and RColorBrewer (v1.0) were used.

Growth experiments

Nine predicted steroid degrading strains, *Pseudomonas resinovorans* NRBC106553, *Sphingomonas wittichii* RW1 (DSM 6014), *Cupriavidus necator* ATCC17699, *Shewanella pealeana* ATCC700345, *Thermomonospora curvata* ATCC19995, *Actinoplanes missouriensis* 431 (ATCC14538), *Salinispora arenicola* CNS-205, *Amycolicicoccus subflavus* DQS3-9A1T and *Amycolatopsis* sp. strain ATCC39116 were used in growth experiments. Growth experiments were carried out with testosterone (1 mM), cholesterol (1 or 0.5 mM) or cholate (0.5 mM) as substrates. Testosterone and cholesterol were (a) added directly to sterile incubation flasks from a stock solution in 2-propanol, after which the 2-propanol was evaporated and sterile medium was added, or (b) added as a solid to medium additionally containing 1 % (w/v) methyl-β-cyclodextrin prior to autoclaving. Cholesterol was alternatively (c) dissolved in tyloxapol (0.5 % v/v) and added to medium prior to autoclaving. The addition of tyloxapol or cyclodextrin was shown to enable or enhance steroid degradation by some bacteria (1, 2), presumably by making steroids more bioavailable. Cholate was added to autoclaved medium from a sterile stock solution.

To test growth on steroids, non-marine strains (NRBC106553, RW1, ATCC17699, ATCC19995, ATCC39116 and 431) were grown in M9 mineral medium supplemented with

trace elements and vitamin B1 plus a steroid substrate. To test metabolism of steroids, the non-marine strains were grown in 50% LB medium or M9 medium supplemented with 0.18 % (v/v) glycerol plus a steroid substrate. To test growth on or metabolism of steroids, marine strains were grown in 50% LB medium supplemented with 22 g l^{-1} sea salts (Instant Ocean Sea Salt, Aquarium Systems Inc., Blacksburg, VA, USA) or in marine broth medium (Difco) plus a steroid substrate. All cultures were incubated at 30°C with shaking, except ATCC19995, which was incubated at 50°C. Growth was monitored as protein, using the bicinchoninic acid assay (Thermo Scientific) after hot alkaline cell lysis. Substrate removal and metabolite production were monitored by gas chromatography-coupled mass spectrometry (GC-MS) of organic extracts of acidified cultures. For all media, uninoculated controls verified sterility and stability of the steroids. For each strain on each medium, controls without steroids were used to quantify any background growth (as on cyclodextrin or tyloxapol), which was subtracted to quantify growth on the steroids. Strains were considered to grow on a steroid when there was an increase in protein compared to control cultures without the steroid added and when the steroid substrate was completely removed and no transformation products were detected by GC-MS analysis. Strains were considered to transform a steroid when transformation products accumulated in the cultures as determined by GC-MS analysis.

1. **Donova MV**, **Nikolayeva VM**, **Dovbnya DV**, **Gulevskaya SA**, **Suzina NE**. 2007. Methyl-beta-cyclodextrin alters growth, activity and cell envelope features of sterol-transforming mycobacteria. **153**:1981–1992.

2. **van der Geize R**, **Yam K**, **Heuser T**, **Wilbrink MH**, **Hara H**, **Anderton MC**, **Sim E**, **Dijkhuizen L**, **Davies JE**, **Mohn WW**, **Eltis LD**. 2007. A gene cluster encoding cholesterol catabolism in a soil actinomycete provides insight into *Mycobacterium tuberculosis* survival in macrophages. Proc Natl Acad Sci USA **104**:1947–1952.
